# A self-healable and highly stretchable supercapacitor based on a dual crosslinked polyelectrolyte

**DOI:** 10.1038/ncomms10310

**Published:** 2015-12-22

**Authors:** Yan Huang, Ming Zhong, Yang Huang, Minshen Zhu, Zengxia Pei, Zifeng Wang, Qi Xue, Xuming Xie, Chunyi Zhi

**Affiliations:** 1 Department of Physics and Materials Science, City University of Hong Kong, Hong Kong 999077, China; 2 Laboratory of Advanced Materials (MOE), Department of Chemical Engineering, Tsinghua University, Beijing 100084, China; 3 Shenzhen Research Institute, City University of Hong Kong, Shenzhen 518000, China

## Abstract

Superior self-healability and stretchability are critical elements for the practical wide-scale adoption of personalized electronics such as portable and wearable energy storage devices. However, the low healing efficiency of self-healable supercapacitors and the small strain of stretchable supercapacitors are fundamentally limited by conventional polyvinyl alcohol-based acidic electrolytes, which are intrinsically neither self-healable nor highly stretchable. Here we report an electrolyte comprising polyacrylic acid dual crosslinked by hydrogen bonding and vinyl hybrid silica nanoparticles, which displays all superior functions and provides a solution to the intrinsic self-healability and high stretchability problems of a supercapacitor. Supercapacitors with this electrolyte are non-autonomic self-healable, retaining the capacitance completely even after 20 cycles of breaking/healing. These supercapacitors are stretched up to 600% strain with enhanced performance using a designed facile electrode fabrication procedure.

## Abstract (web summary)

Materials for wearable energy storage devices should be mechanically durable. Here, the authors report a supercapacitor composed of polyacrylic acid dual cross-linked by hydrogen bonding and vinyl hybrid silica nanoparticles which is self-healable and retains performance when stretched up to 600%.

## Graphical abstract

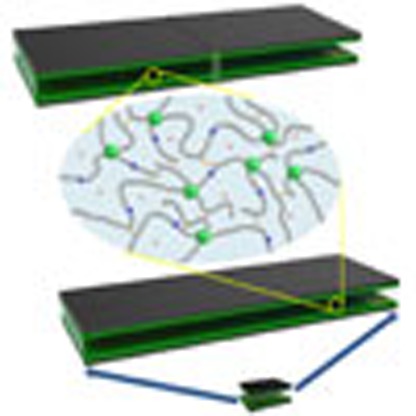

The design of highly self-healable and stretchable devices is a radical element in the development of many unprecedented applications such as electronic skin^1^,^2^ and smart energy storage clothes^3^,^4^. Portable and wearable supercapacitors, coupled with either self-healability^5^,^6^ or stretchability^7^, have particularly become one mainstream in personalized electronics for their high-power density, fast rate of charge–discharge and long cycling lifetime in addition to the aforementioned functions^8^,^9^,^10^,^11^,^12^. On one hand, self-mending polymeric materials based on physical crosslinking^13^,^14^ or external stimuli such as heat^15^,^16^ and light^17^,^18^, have been successfully developed and employed for the mechanical recovery and electrical restoration of devices^5^,^6^,^19^. Besides an indispensable layer of electrolyte sandwiched between two electrodes, an extra layer of self-healing polymer is wrapped onto electrodes or used as a substrate for all self-healable supercapacitors reported thus far. However, a key constraint lies in their low healing efficiency and cyclability. After merely a few breaking/healing cycles (no more than 5), the capacitances decrease dramatically (14.3–28.2%)^5^,^6^,^19^. Another desirable feature missing in these devices is volume/mass economy owing to the use of an additional component, lowering the volumetric/mass capacitance. On the other hand, given that all electrocapacitive materials are not intrinsically stretchable, various modified structures (for example, non-coplanar buckled^20^, coplanar serpentine and wavy^21^,^22^, percolating nanostructured^23^) and electron/ion-inactive stretchable substrates (such as elastomers^24^,^25^ and stretchable textiles^26^) have been utilized to introduce stretchability into conventionally rigid supercapacitors. However, most achieved strains did not exceed 100% (refs 24, 27, 28) and the performance usually deteriorated at super-high strains^29^. All these limitations of self-healable and stretchable supercapacitors are fundamentally attributed to the fact that the widely used polyvinyl alcohol (PVA)-based acidic electrolytes are neither healable nor very stretchable, giving rise to disadvantages of unsatisfactory performance, additional components and complex multi-step designs.

Therefore, it is primarily important to develop a multifunctional polyelectrolyte to realize intrinsic self-healability and high stretchability. This requires effective ionic conduction, efficient crack-activated hydrogen bonds crosslinking and reversible crosslinking interactions among polymer chains. Compared with the extensive and substantial research on electrodes, fewer relevant studies on indispensable polyelectrolytes have been performed, and even fewer studies have been conducted on multifunctional polyelectrolytes. In this paper, a new electrolyte is developed comprising polyacrylic acid dual crosslinked by hydrogen bonding and vinyl hybrid silica nanoparticles (VSNPs-PAA). The prepared polyelectrolyte possesses all these advantages of tuneable ionic conductivity, self-healability and high stretchability. Movable protons in the polyelectrolyte provide an equivalent electrode capacitance compared with the commonly used PVA/H_3_PO_4_ electrolyte^4^,^12^,^30^,^31^. It can be easily self-repaired at room temperature and repaired samples display ionic properties similar to those of pristine samples after cycles of breaking/healing. In addition, it can be stretched over 3,700% without any crack, suggesting effectively reversible crosslinking interactions through stress transfer and energy dissipation. Supercapacitors are facilely assembled by the use of this polyelectrolyte and polypyrrole (PPy)-deposited carbon nanotube (CNT) paper electrodes with the incorporation of small CNT patches on the cutting wounds and a pre-stretched wavy structure. They exhibit the merits of self-healability (∼100% efficiency during all 20 breaking/healing cycles), stretchability (600% strain with enhanced performance), the fewest number of components and facile fabrication.

## Results

### Synthesis and physicochemical properties of VSNPs-PAA

**Figure 1:**
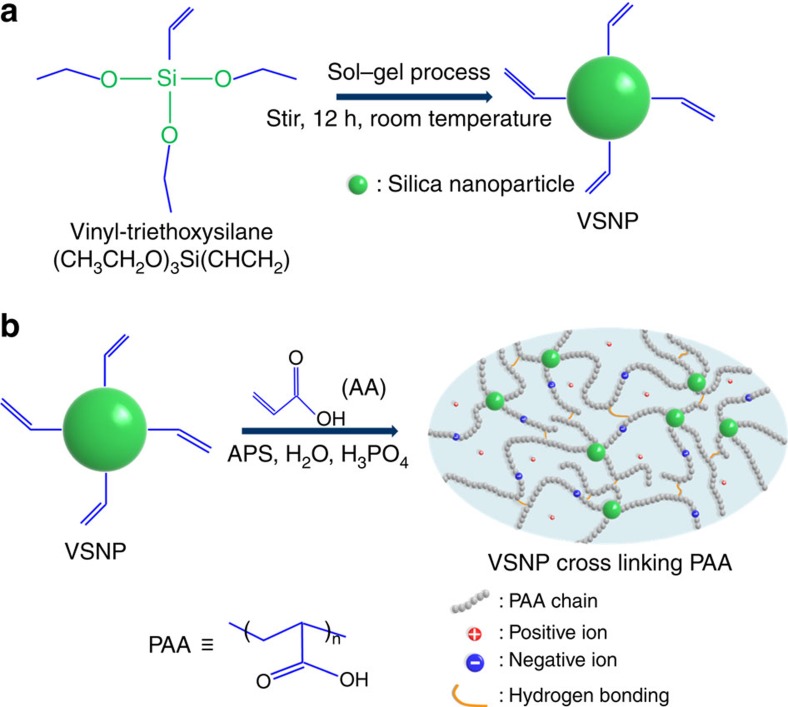
Preparation of the polyelectrolyte by using VSNPs crosslinker. (**a**) Preparation of VSNPs from vinyltriethoxysilane. (**b**) Preparation of the VSNPs-PAA electrolyte from VSNPs (crosslinker), acrylic acid (AA, main monomer), ammonium persulfate (APS, initiator) and phosphoric acid (pH and water content regulator).

Silica nanoparticles-based nanocomposite polymers are widely employed to reinforce mechanical properties of polymeric materials^32^,^33^. We used a sol–gel method for the preparation of VSNPs^34^,^35^ (Fig. 1a; Supplementary Fig. 1a). An acrylic acid monomer and the as-prepared VSNPs (Supplementary Fig. 1b,c) were polymerized together in the presence of ammonium persulfate as the initiator. Phosphoric acid served as the regulator of water and proton ion content (Fig. 1b). By doing so, we took advantage of synergistic effects as follows. First, VSNPs serve as covalent crosslinking points and stress transfer centres, thus strengthening the network structure under large strains. Second, the PAA polymer chains provide sufficient intra- and intermolecular hydrogen bonding. The non-sacrificial hydrogen bond crosslinking is crucial for self-healing. Moreover, the broken intermolecular hydrogen bonds can dynamically recombine to dissipate energy and homogenize the network under stretching. Third, the variable content of water and protons in the VSNPs-PAA can tune the ionic conductivity, making the synthesized polymer an excellent candidate as the electrolyte of supercapacitors. These synergistic effects are responsible for the multiple functions of high stretchability and self-healability observed from our polyelectrolyte.

**Figure 2:**
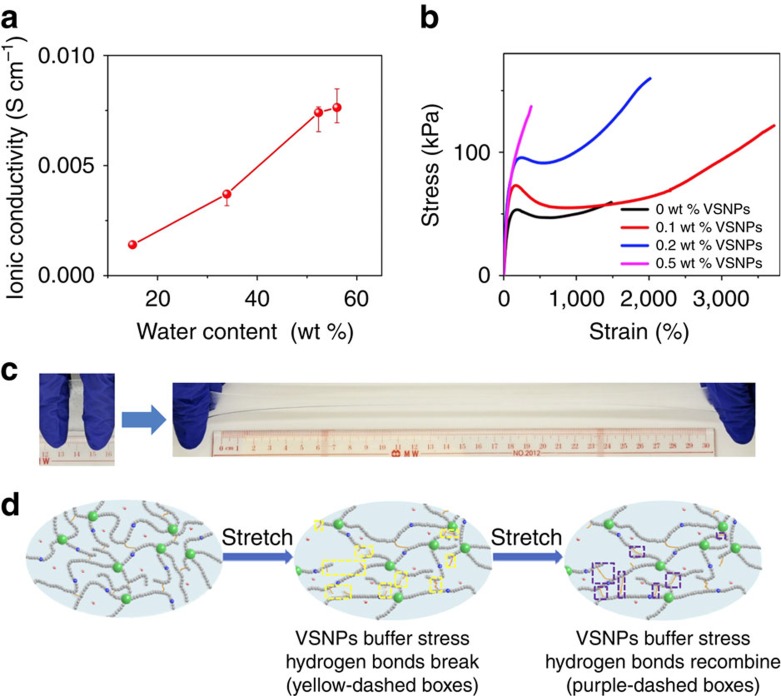
Physicochemical properties of the VSNPs-PAA electrolyte. (**a**) Ionic conductivity as a function of water content in the VSNPs-PAA (weight fraction of water in the gel). (Error bars in the first two points are too small to be observed, and they are based on maximum and minimum values in five measurements.) (**b**) Stress–strain curves of VSNPs-PAA (60 wt% H_2_O) with various contents of VSNPs under stretching. (**c**) Relaxed (left of panel) and elongated (right of panel) state of the VSNPs-PAA, exhibiting excellent stretchability. (**d**) Schematic of the origin of super stretchability.

With the increase in the water content, ions in the VSNPs-PAA move more easily, and the VSNPs-PAA chains are more sufficiently extended. Therefore, the ionic conductivity increases remarkably with increasing water content and is comparable to the conventional PVA/H_3_PO_4_ electrolyte^36^ (Fig. 2a), suggesting the use of VSNPs-PAA as the electrolyte of supercapacitors. The content of VSNPs indicates the density of crosslinking points, which strongly influences the mechanical properties (Fig. 2b). Compared with pure PAA, a low VSNPs content (0.1 wt%) substantially increases the strength and the stretchability to >3,700% strain (Fig. 2b,c), which is much higher than those stretchable polymers^37^,^38^,^39^,^40^,^41^. A more detailed analysis of the effect of VSNPs content on mechanical properties is provided in Supplementary Fig. 2. As mentioned above, the super-high stretchability arises from the VSNPs-aided toughening and hydrogen bond crosslinking. General polymers have a randomly coiled conformation in a relaxed state. Once the imposed strain achieves a specific extent, it becomes difficult to uncoil the polymer chains. The energy can then be dissipated only through the rupture of the entangled polymer chains. In the case of our VSNPs-PAA, VSNPs and hydrogen bonding are the primary crosslinking mechanisms other than the polymer chain entanglement. The propagation of cracks is delayed by dispersing the applied stress via the anchoring VSNPs, resulting in the high strains observed. Benefited from the intermolecular hydrogen bonds as reversible physical crosslinking points, our VSNPs-PAA can dynamically break and recombine to dissipate energy, as schematically shown in Fig. 2d. This dynamic process reorganizes the polymer chains and thus distributes the applied stress rapidly and uniformly over the entire network.

**Figure 3:**
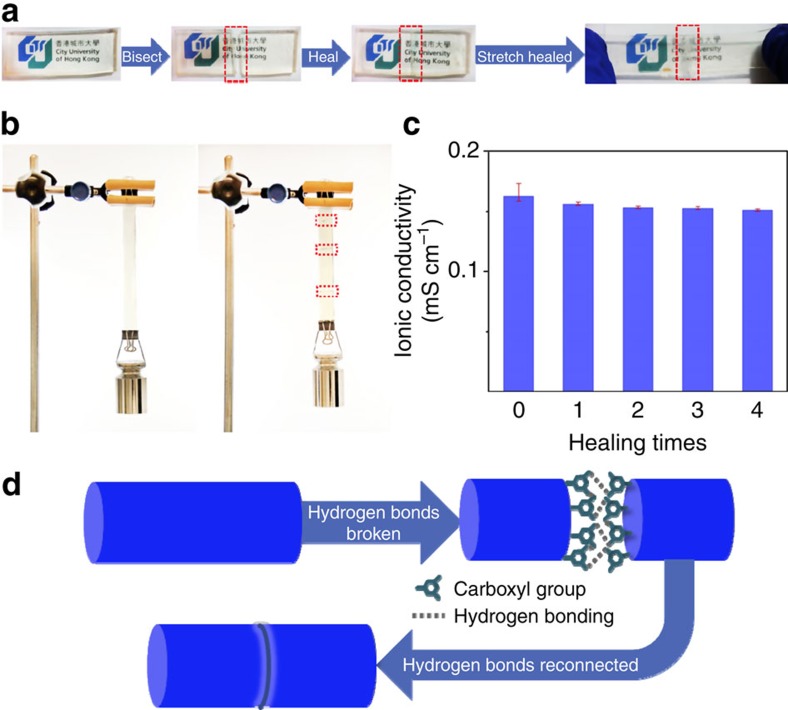
Self-healing properties of the VSNPs-PAA electrolyte. (**a**) Demonstration of the self-healing process of VSNPs-PAA. (**b**) Demonstration of the self-healed VSNPs-PAA (3.4 mm thick and 1.8 cm wide) to completely support ca. 500 g mass, which equates to 80 kPa of stress: pristine sample (left of panel) and healed sample after the third breaking/healing cycle (right of panel). Red rectangles indicate the wound/healing positions. (**c**) Ionic conductivity of the VSNPs-PAA after multiple breaking/healing cycles. (Error bars are based on maximum and minimum values in five measurements.) (**d**) Schematic of self-healing arising from interfacial hydrogen bonding.

The intermolecular hydrogen bonds among the crosslinked polymer chains on the VSNPs are also responsible for the superior self-healing property demonstrated in Fig. 3a. First, a VSNPs-PAA belt was bisected with scissors. The fresh cuts were then brought into contact under mild pressure. After the breakage was successfully self-healed within tens of minutes in the ambient condition, the belt could be stretched extensively without breaking. This good self-healing performance enabled our VSNPs-PAA to fulfil the demanding mechanical requirements (Fig. 3b). After a couple of breaking/healing cycles, the ionic conducting properties were well restored (Fig. 3c), and decent mechanical performance was maintained (Supplementary Fig. 3). To investigate the self-healing mechanism, a 1 M aqueous solution of urea (an efficient hydrogen-bond-breaking reagent^42^,^43^) was used to prevent the formation of hydrogen bonds in the wound area (Supplementary Methods). The healed urea-treated VSNPs-PAA had much lower strain, tensile strength and tensile modulus, compared with the healed VSNPs-PAA without the urea treatment (Supplementary Fig. 4). This indicates that the self-healing was remarkably weakened and reveals that the self-healing was dominated by the formation of abundant reversible intermolecular hydrogen bond crosslinks^13^,^14^. When breakage occurs in the contact region, the broken hydrogen bonds can recombine through the coordination of carboxyl groups on the PAA main chains (Fig. 3d).

### Electrochemical characterization of VSNPs-PAA

**Figure 4:**
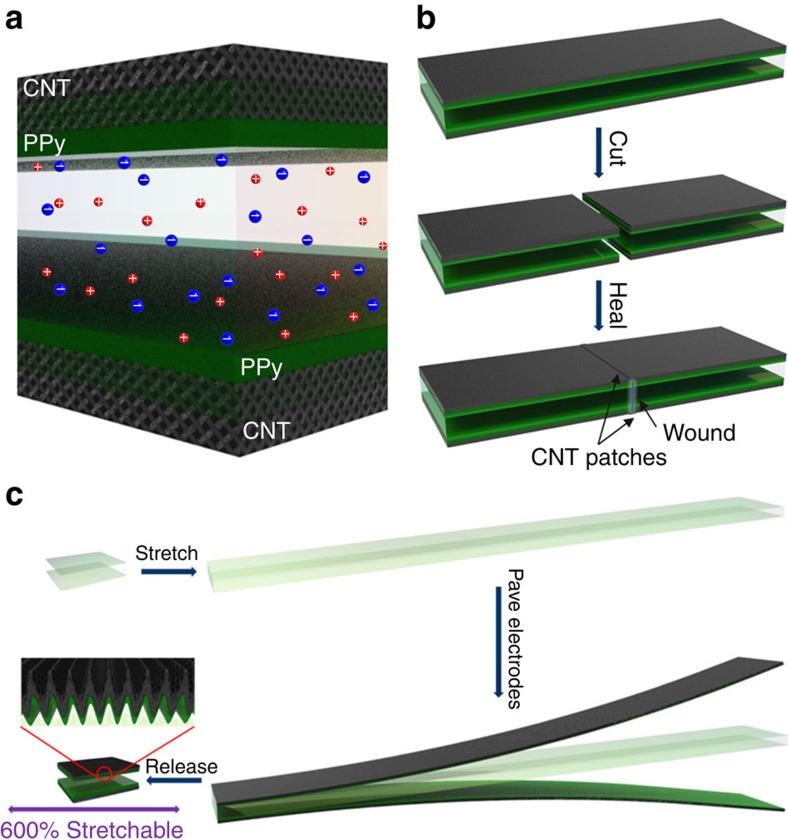
Schematics of fabrication strategies for specific functional supercapacitors. (**a**) Schematic of the supercapacitor comprising the VSNPs-PAA polyelectrolyte and PPy@CNT paper electrodes. (**b**) Fabrication of the patch-assisted non-autonomic self-healable supercapacitor. (**c**) Schematic of the fabrication of a super-stretchable supercapacitor.

The synthesized VSNPs-PAA film was used as an electrolyte without further treatment. PPy electrodeposited on CNT papers were utilized as both active materials and current collectors to construct solid-state supercapacitors. Besides its contribution to capacitance, the flexible PPy can serve as a stress buffer during stretching, which is discussed below. Typically, two PPy@CNT paper electrodes were directly paved on the VSNPs-PAA film electrolyte without a binder or separator (Fig. 4a). For the fabrication of a self-healable supercapacitor, small patches of conductive CNT paper were facilely paved on wounds to completely restore the electrical conductivity (Fig. 4b). Regarding the fabrication of a stretchable supercapacitor, the VSNPs-PAA polyelectrolyte film was first pre-stretched to over 600% strain. The PPy@CNT papers were then paved on each side of the stretched electrolyte layer. After releasing, the stretchable supercapacitor was realized with a wavy electrode structure (Fig. 4c).

**Figure 5:**
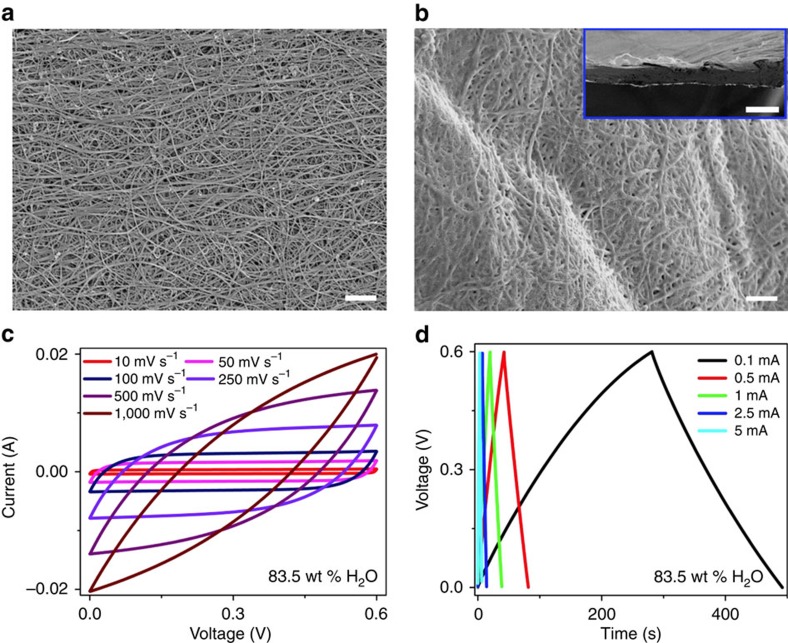
Electrochemical performance of the VSNPs-PAA polyelectrolyte. (**a**) An SEM image of the CNT paper. Scale bar, 1 μm. (**b**) An SEM image of the PPy electrodeposited on CNT paper. Scale bar, 1 μm. (Inset is a cross-sectional SEM image of the PPy electrodeposited on CNT paper. Scale bar, 50 μm.) (**c**) CV curves at various scan rates from 10 to 1,000 mV s^−1^. (**d**) GCD curves at various charging/discharging currents from 0.1 to 5 mA.

Figure 5a shows that CNT papers have a typical morphology of interweaved nanowires. By contrast, a thin film of PPy was uniformly electrodeposited on CNT papers (Fig. 5b). The PPy-deposited CNT electrode was intentionally folded before the scanning electron microscopy (SEM) observation. Notably, the electrode was not cracked because of the good flexibility of both PPy and the CNT paper. The prepared species was confirmed by Raman spectroscopy (Supplementary Fig. 5). The PPy has a thickness of <90 nm (Fig. 5b), favouring rapid ion transportation during charge/discharge.

The cyclic voltammetry (CV) curves at scan rates of up to 1,000 mV s^−1^ and galvanostatic charge/discharge (GCD) curves at various currents from 0.1 to 5 mA are shown in Fig. 5c,d. Notably, the scan rates achieved here are much higher than most rates of PPy-based electrodes measured even in aqueous electrolytes^44^,^45^, and CVs maintain the rectangular shape at a high scan rate of 250 mV s^−1^. These results indicate that the supercapacitor can endure very high voltage/current change rates, which is believed to be a result of the excellent ionic conductivity of the polyelectrolyte and effective electrochemical dynamic processes in the electrodes. At higher scan rates of 500 and 1,000 mV s^−1^, CVs deviate from the rectangular shape and gradually display more features of a resistor^46^,^47^. This trend mainly arises from the higher ionic transfer resistance at the higher scan rate/current owing to the diffusion limitation^48^,^49^. The capacitances were calculated using both these CV and GCD curves (Supplementary Fig. 6), which are comparable to or even higher than results tested in liquid electrolytes^26^,^44^,^50^,^51^,^52^,^53^. With the same mass ratio of the electrolyte composition, electrocapacitive curves of PPy supercapacitors developed using our VSNPs-PAA completely overlay those generated using the common PVA (Supplementary Fig. 7). This result suggests that VSNPs-PAA can be a perfect alternative to PVA as the electrolyte without compromising electrode performance. The electrochemical performance of the VSNPs-PAA polyelectrolyte varies with the water content in the polyelectrolyte (Supplementary Fig. 8) but is independent of the VSNPs content (Supplementary Fig. 9).

### Complete self-healability of the supercapacitor

The self-healable VSNPs-PAA polyelectrolyte promises the mechanical and electronic self-healing properties of the supercapacitor. Small patches of CNT paper are utilized to overcome the well-known misalignment problem of self-healing supercapacitors. SEM images of both the polyelectrolyte and the PPy@CNT electrode before and after healing are provided in Supplementary Figs 10 and 11.

**Figure 6:**
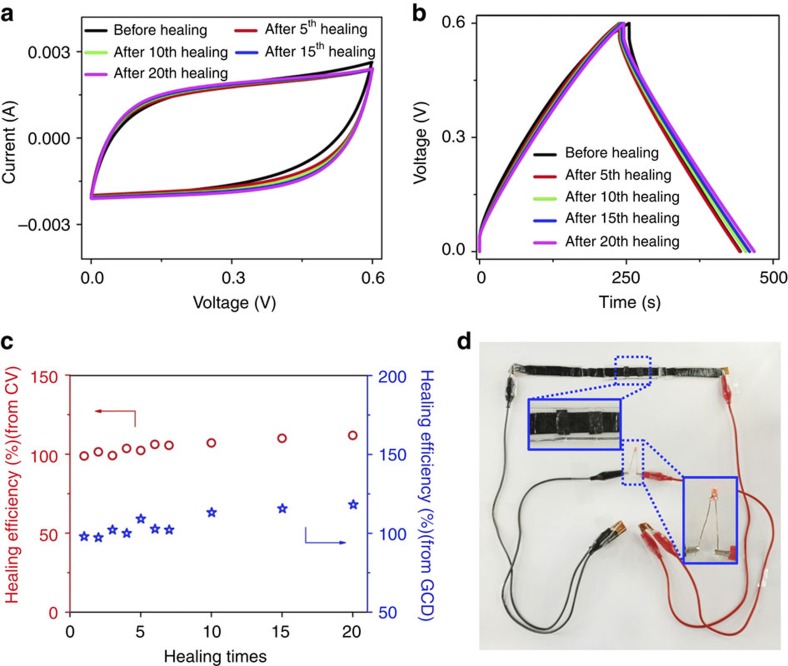
Self-healing performance of the supercapacitor comprising the VSNPs-PAA polyelectrolyte. (**a**) CV curves at a scan rate of 5 mV s^−1^. (**b**) GCD curves at a charging/discharging current of 1 mA. (**c**) Healing efficiency calculated from CV (circle, red) and GCD (star, blue) curves. (**d**) A photo of three supercapacitors connected in series, one of which has self-healed twice to power an LED bulb after self-healing. (Insets show enlarged profiles of the twice self-healed supercapacitor and the lit LED bulb.)

Electrochemical performances of the patch-assisted non-autonomic self-healable supercapacitor were systematically investigated. Remarkably, both CV and GCD curves overlay almost completely even after the 20th breaking/healing cycle (Fig. 6a,b). As shown in Fig. 6c, the healing efficiency is ∼100% during all breaking/healing cycles, extremely outperforming other reported self-healable devices (no more than five cycles, 14.3 to 28.2% performance degradation)^5^,^6^,^19^ (Supplementary Fig. 12). These results reveal the superiority of adopting our intrinsic self-healable electrolyte over conventional methods, which use additional outer or inner self-healable components to realize the self-healing^5^,^6^,^19^. This excellent self-healing property is also attributed to the high electrical conductivity of CNT paper patches, which connect the broken parts effectively (Supplementary Fig. 13). Notably, the healing efficiency slightly fluctuates near 100% in these breaking/healing cycles. This could be explained by the accidental micro-adjustment between the broken electrodes. That is, the macroscopic manual operation would cause occasional microcosmic adjustment of the reconnected electrodes, leading to slight fluctuation of the performance during breaking/healing cycles. In sum, it is the utilization of an intrinsically self-healable electrolyte and small CNT patches that provides our supercapacitor with such extreme self-healability, which is attractive for high-performance self-healable devices. Note that the fabrication of highly self-healable supercapacitors is very facile and effective (Fig. 6d, Supplementary Note 1 and Supplementary Movie 1), thus facilitates the future wide-scale adoption of these supercapacitors.

### Highly stretchable supercapacitor

**Figure 7:**
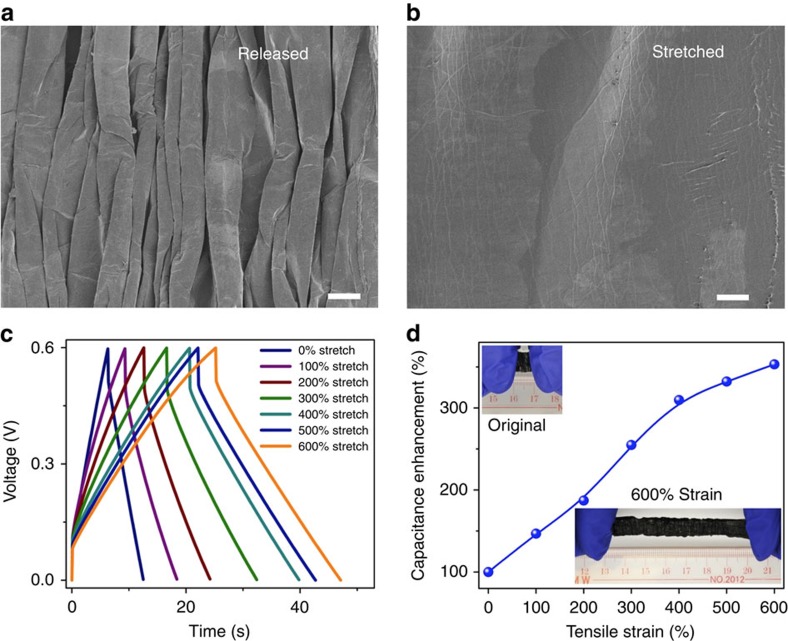
Electrochemical performance of the supercapacitor comprising the VSNPs-PAA polyelectrolyte under high stretching. (**a**) An SEM image of the released PPy@CNT paper electrode after pre-stretching. Scale bar, 50 μm. (**b**) An SEM image of the stretched PPy@CNT paper electrode. Scale bar, 50 μm. (**c**) GCD curves from 0 to 600% strain at a charging/discharging current of 2.5 mA. (**d**) Capacitance enhancement ratio obtained from GCD curves as a function of the tensile strain. (Insets are photos of the supercapacitor at a fully released state and 600% strain.)

Pre-strain is an effective approach to fabricate stretchable/highly stretchable devices^7^,^41^,^54^. By using this approach, the supercapacitor achieved a high strain of 600% and electrodes were stably attached to the electrolyte. SEM images of the polyelectrolyte before and after stretching are shown in Supplementary Fig. 14. Owing to the excellent flexibility of the PPy and CNT paper^47^,^55^,^56^, no cracks on the released and stretched electrodes were observed from the SEM image (Fig. 7a,b).

The wavy structure avoids the disadvantage of general stretchable devices, the performance of which is usually limited by the structural breakdown arising from the stretch imposed. By contrast, the fabricated supercapacitor exhibits enhanced electrochemical performance under stretching (Fig. 7c; Supplementary Fig. 15a). Both GCD and CV profiles expand with the increased strain. The capacitance calculated from the GCD and CV curves achieves an increase of 3.5-fold and 2.1-fold at 600% strain, respectively (Fig. 7d and Supplementary Fig. 15b). To the best of our knowledge, such device-level high intrinsic strain has never been achieved. The increased capacitance is a result of the larger contact areas between electrodes and the electrolyte induced by stretching. As seen in Figs 4c and 7a, there are uncontacted areas on the free wavy electrodes. Upon stretching, some initially uncontacted parts contact with the electrolyte, thus providing more effective electrode materials to participate in the electrocapacitive process. Because the highly flexible electrodes are paved following the pre-stretching of the electrolyte, no structural breakdown is induced by the subsequent applied stretching. Therefore, it is the pre-stretched structure and the utilization of highly flexible PPy@CNT paper electrodes that provide our supercapacitor with enhanced capacitance under super strains, which is attractive for high-performance super-stretchable devices. Note that although polydimethylsiloxane is a common substrate material used in super-stretchable devices, it cannot fulfil the function as an electrolyte due to the lack of free-moving ions (Supplementary Fig. 16). As a demonstration, our supercapacitor effectively powers an LED bulb during stretching. The complete process is shown in the Supplementary Movie 2. Similar to stretching, electrochemical performance is also improved by the compressive strains (Supplementary Fig. 17). This mainly arises from the improved interfacial contact and thus the ion transfer from the electrolyte to the surface of the electrode, rather than the thinner electrolyte (Supplementary Fig. 18).

The excellent self-healability and high stretchability validate the application as multifunctional supercapacitors. To the best of our knowledge, the integration of these functions into one supercapacitor has never been reported, not to mention the facile assembly and the fewest number of components achieved in this work.

## Discussion

A multifunctional polyelectrolyte was fabricated by polyacrylic acid dual crosslinked by hydrogen bonding and vinyl hybrid silica nanoparticles. The polyelectrolyte can be easily stretched to greater than 3,700% strain and, once cut, can be simply self-healed by combing the broken interfaces in ambient conditions. Benefited from the intrinsic stretchability, self-healability and ionic conductivity of the new polyelectrolyte, supercapacitors based on this polyelectrolyte are highly superior to those with conventional PVA-based acidic electrolytes. For example, the facilely fabricated supercapacitor possesses intrinsic self-healability and the capacitance is maintained completely during all 20 breaking/healing cycles. This significantly outperforms all reported self-healable devices. In addition, it can be 600% stretchable by simply incorporating wavy electrodes. To the best of our knowledge, the self-healing efficiency and stretchability of supercapacitor devices achieved here are the best among all that have been reported, which originate from the unique polyelectrolyte structure of polyacrylic acid dual crosslinked by hydrogen bonding and vinyl hybrid silica nanoparticles. Our study of the superior multifunctionality at the device level with facile fabrication and the fewest components creates considerable potential for the wide-scale application of multifunctional devices in many fields such as energy storage and biomimetic sensing.

## Methods

### Synthesis of vinyl hybrid silica nanoparticles-crosslinked polyacrylic acid electrolyte

First, vinyltriethoxysilane (3.8 g, Alfa Asear) was added into de-ionized water (30 g) under vigorous stirring until the oil-like droplets completely disappeared and a transparent dispersion of vinyl hybrid silica nanoparticles was obtained (12 h). An acrylic acid monomer (12 g, Beijing Chemical Reagent) and ammonium persulfate (0.024 g, Xilong Chemical) were then added into the diluted aqueous dispersion of vinyl hybrid silica nanoparticles (18 ml, 0.067 wt%) and stirred for 0.5 h at room temperature. Before use, the acrylic acid monomer was purified by distillation under reduced pressure and stored in a refrigerator. After the magnetic stirring, the solution was degassed and sealed under N_2_ to remove the dissolved oxygen. Next, free-radical polymerization was allowed to proceed in a water bath at 40±2 °C for 30 h. Finally, the as-prepared polymer was fully dried at room temperature and then soaked in phosphoric acid (500 ml) at various concentrations (0, 5, 10, 20, 30, 40, 50, 60 and 70 wt%) for 3 weeks to achieve the equilibrated state.

### Fabrication and electrochemical characterization of the supercapacitor using VSNPs-PAA as the polyelectrolyte

CNT papers (width: 1 cm; length: 20 cm) were electrodeposited with PPy at 0.8 V versus Ag/AgCl for 10 min in a solution of 0.1 M *p*-Toluenesulfonic acid, 0.3 M sodium toluenesulfate and 0.5% pyrrole monomer (v:v) at 0 °C. Before electrodeposition, pyrrole was distilled to purify the pyrrole monomers. Two identical deposited PPy@CNT papers were placed on each side of the VSNPs-PAA film under ambient conditions. Thus, the solid-state supercapacitor was obtained with the electrolyte also serving as a separator. The performance of the assembled supercapacitors was measured by CV and GCD in a two-electrode configuration using a potentiostat (CHI 760E). Electrochemical impedance spectra (EIS) were measured at frequencies ranging from 0.01 to 5,000 Hz with a potential amplitude of 5 mV. All measurements were performed at room temperature. The capacitance with respect to the single electrode (*C*_*m*_) was calculated using the charge integrated from GCD and CV curves individually according to the following formulas:


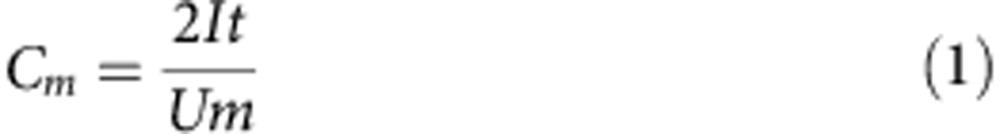



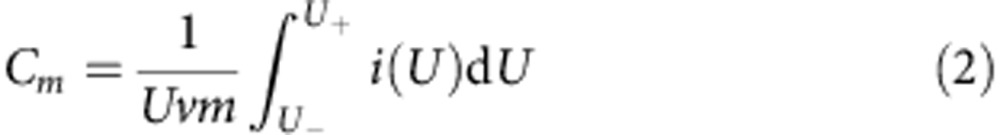


where *I* is the discharge current during GCD, *t* is the discharge time during GCD, *U* is the voltage range (*U*=*U*_+_−*U*_−_), *m* is the mass of PPy on one electrode, *v* is the scan rate of the CV curve and *i*(*U*) is the current during CV.

## Additional information

**How to cite this article:** Huang, Y. *et al*. A self-healable and highly stretchable supercapacitor based on a dual crosslinked polyelectrolyte. *Nat. Commun.* 6:10310 doi: 10.1038/ncomms10310 (2015).

## Supplementary Material

Supplementary Information Supplementary Figures 1-18, Supplementary Note 1, Supplementary Methods and Supplementary References

Supplementary Movie 1 The supercapacitor self-healing.

Supplementary Movie 2 The supercapacitor stretching.
