# Supplementary material for: A self-healable and highly stretchable supercapacitor based on a dual crosslinked polyelectrolyte
Source: Nat Commun. 2015 Dec 22;6:10310. doi: 10.1038/ncomms10310 (PMC4703889; doi:10.1038/ncomms10310)
Supplement: Supplementary Information — Supplementary Figures 1-18, Supplementary Note 1, Supplementary Methods and Supplementary References [file ncomms10310-s1.pdf]

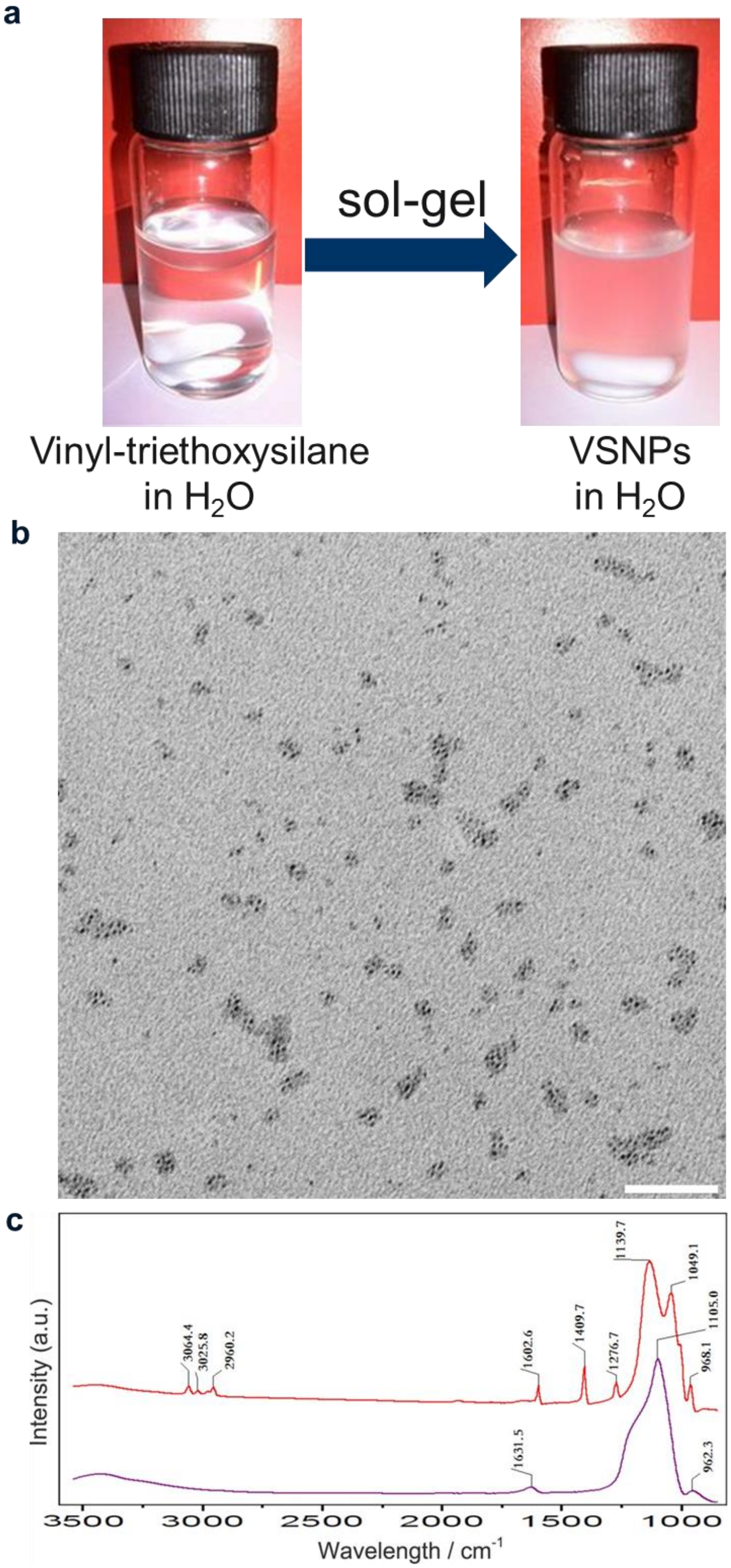
a
sol-gel
Vinyl-triethoxysilane
in H_2O
VSNPs
in H_2O
b
c
3064.4
3025.8
2960.2
1602.6
1409.7
1276.7
1139.7
1049.1
1105.0
968.1
1631.5
962.3
Intensity (a.u.)
3500
3000
2500
2000
1500
1000
Wavelength / cm^{-1}

Supplementary Figure 1 | Characterization of vinyl hybrid silica nanoparticles (VSNPs). a, Images of the synthesis of VSNPs: before (left) and after (right) the sol-gel process. **b**, Transmission electron microscope (TEM) image of highly monodispersed VSNPs with an average diameter of 3 nm. Scale bar: 100 nm. **c**, Fourier transform infrared (FTIR) spectrum of SNPs (purple) and VSNPs (red) revealing vinyl groups dangling on the surface of VSNPs. Before the sol-gel process, an oil-like layer formed when vinyltriethoxysilane was added into de-ionized water. A transparent dispersion of VSNPs was obtained after the sol-gel process. The TEM image reveals that the VSNPs are highly monodispersed with an average diameter of 3 nm. As evidenced by the FTIR spectrum analysis, VSNPs are multifunctional silica nanoparticles with a large number of vinyl groups on their surfaces (Supplementary Fig. 1c). The absorption bands at 1602 cm^{-1}, 2960 cm^{-1}, 3025 cm^{-1} and 3064 cm^{-1} are attributed to the presence of vinyl groups ($—CH=CH_2$). SNPs are bare silica nanoparticles with only hydroxyl groups on their surfaces. The vinyl groups of VSNPs can easily copolymerize with acryl acid (AA) to form gelators that have VSNPs and PAA chains as multifunctional cores and flexible shells, respectively.

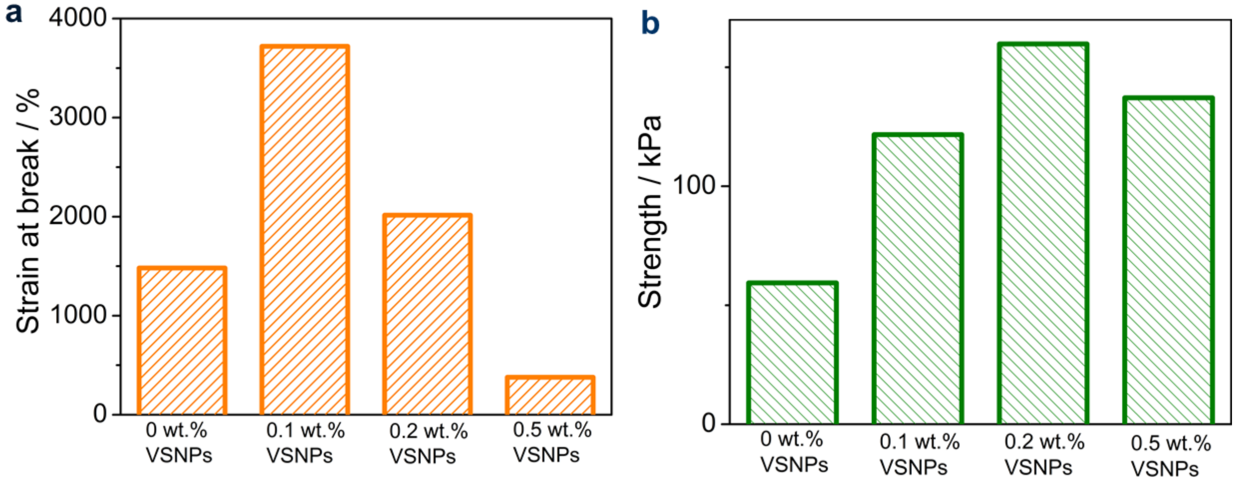

Supplementary Figure 2 | Mechanical properties of VSNPs-PAA with various contents of VSNPs. a, Strain at breaking of VSNPs-PAA (60 wt.% H_2O). **b**, Strength of VSNPs-PAA (60 wt.% H_2O). The content of VSNPs strongly influences the mechanical properties of VSNPs-PAA. Compared with pure PAA, a low content of VSNPs (0.1 wt.%) significantly increases the strain to over 3700%. The fact that strain decreases remarkably with the increase of VSNPs is attributed to a decrease in the average chain length of the cross-linked PAA chains by VSNPs. Tensile strengths of VSNPs-PAA at various VSNPs contents are all higher than that of pure PAA. With an initial increase in VSNPs content, the tensile strength increases. This is explained by the remarkable increase in the density of cross-linking points and by a decrease in the average chain length over a small range. With a further increase in VSNPs content, the density of cross-linking points becomes too high and the average chain length decreases remarkably. This makes the polyelectrolyte brittle and thus, decreasing the tensile strength at higher VSNPs contents.

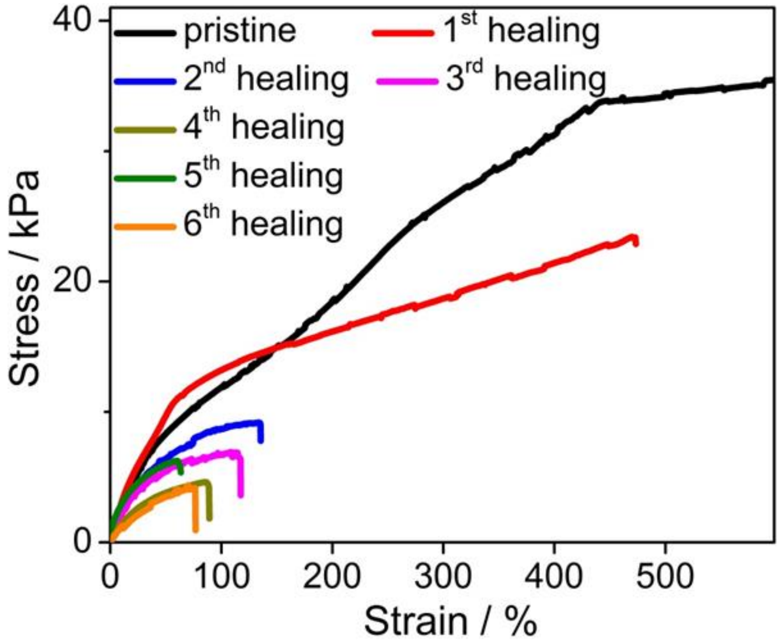

Supplementary Figure 3 | Stress–strain curves for VSNPs-PAA after multiple breaking/healing cycles. The mechanical properties of VSNPs-PAA before and after self-healing were characterized by tensile tests. After the first healing, elongation still reaches ~500%. When the cycle is higher than 4, the elongation fluctuates around 76%, a value still comparable with some polymers. This fluctuation can be explained by the so-called “barrel effect”, by which the healing result depends on the poorest performing cycle, as opposed to the last one, during multiple breaking/healing cycles.

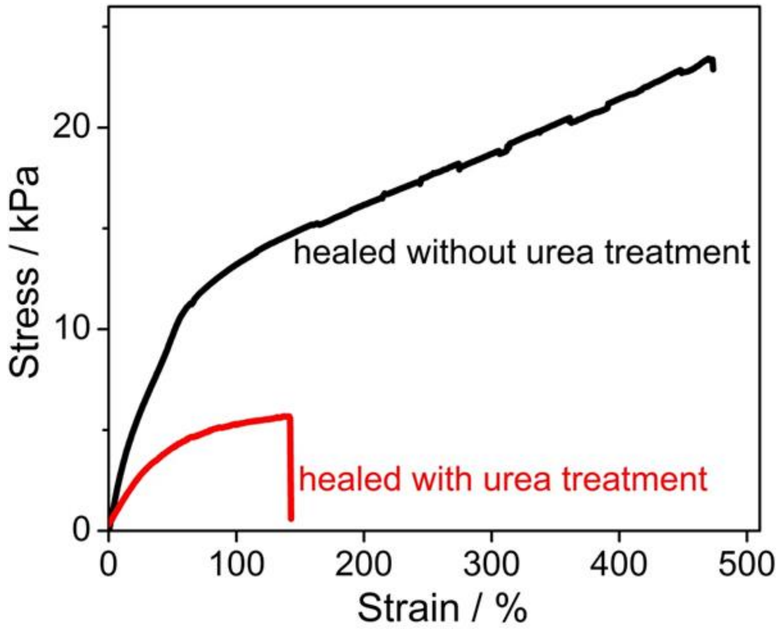

Supplementary Figure 4 | Stress–strain curves for VSNPs-PAA without breaking (black) and with breaking (red) of hydrogen bonds by urea. To reveal the self-healing mechanism of the VSNPs-PAA and verify the proposed hydrogen bonding mechanism, we used urea[1,2] to prevent the formation of hydrogen bonds. The VSNPs-PAA healed following the urea treatment possessed much lower strain, tensile strength, and tensile modulus in comparison with that healed without the treatment. This clearly reveals that hydrogen bonding is the dominant mechanism for the self-healing of VSNPs-PAA materials. Experimental details are as follows: The cut surfaces were coated with several drops of 1 M aqueous solution of urea (an efficient hydrogen-bond-breaking reagent) for 5 minutes. Both the urea-treated and non-urea-treated VSNP-PAAs were healed using identical procedures. Then they were mounted on a Zwick-Roell Z005 machine for tensile tests.

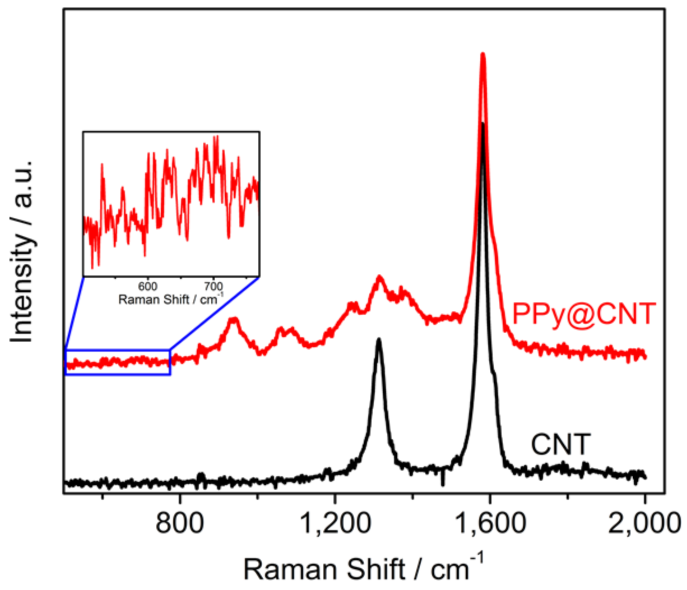

Supplementary Figure 5 | Raman spectra of CNT and PPy (The inset is the zoomed-in spectrum from 500 cm^{-1} to 770 cm^{-1}, showing the Raman bands of PPy around 630 cm^{-1} and 700 cm^{-1}). Two typical peaks of CNT at 1314 cm^{-1} and 1580 cm^{-1} are observed, which correspond to the D band (vibrations of carbon atoms with an sp^3 electronic configuration) and the G band (in-plane vibration of carbon atoms with an sp^2 bond), respectively[3]. The Raman spectrum confirms the species of PPy. The band around 630 cm^{-1} is attributed to ring torsion. The region around 700 cm^{-1} corresponds to C-H wagging. The peak at around 935 cm^{-1} corresponds to ring deformation. The band at 987 cm^{-1} is attributed to ring deformation associated with dictation. The peaks at 1059 cm^{-1} and 1092 cm^{-1} correspond to the symmetrical C–H in-plane bending and N–H in-plane deformation. The peaks at 1249 cm^{-1} and 1316 cm^{-1} are attributed to the antisymmetrical C–H in-plane bending and antisymmetrical in-ring C–N stretching, respectively. C–C and C–N stretching are reflected at both 1382 cm^{-1} and 1506 cm^{-1}. Beside the G band of CNT, the peak at 1580 cm^{-1} is an overlap of C–C in-ring and C–C inter-ring stretching resulting from radical cations and dictation[4].

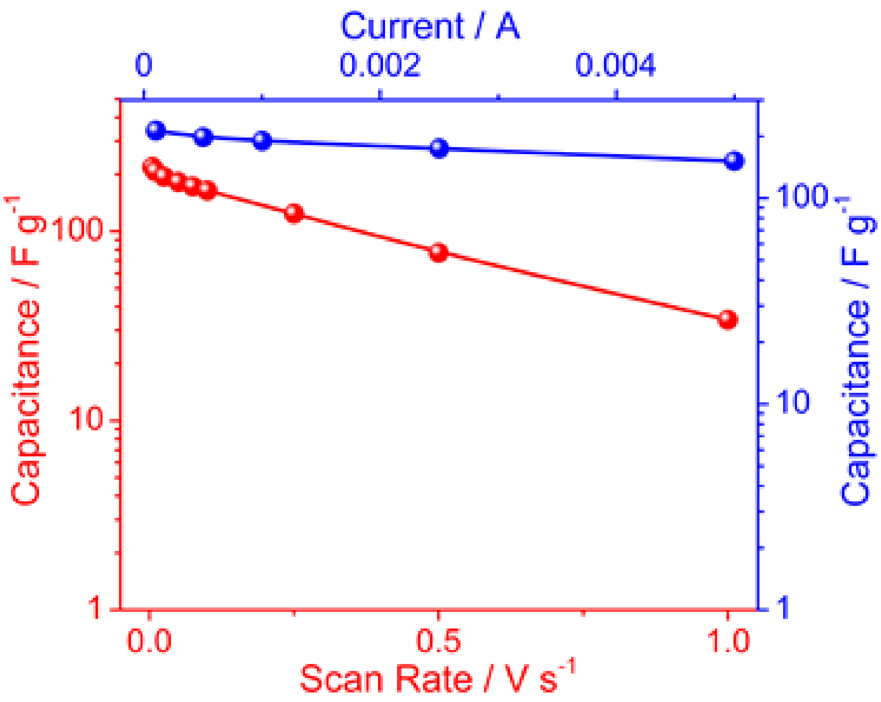

Supplementary Figure 6 | Capacitances calculated from CV (red) and GCD (blue) curves of the PPy@CNT paper electrodes using VSNPs-PAA film polyelectrolyte (83.5 wt.% H_2O). Capacitances calculated from CV (red) and GCD (blue) curves of PPy@CNT paper electrodes using VSNPs-PAA (83.5 wt.% H_2O) as a polyelectrolyte are comparable with and even higher than results with liquid electrolytes. This reveals that VSNPs-PAA is an excellent electrolyte.

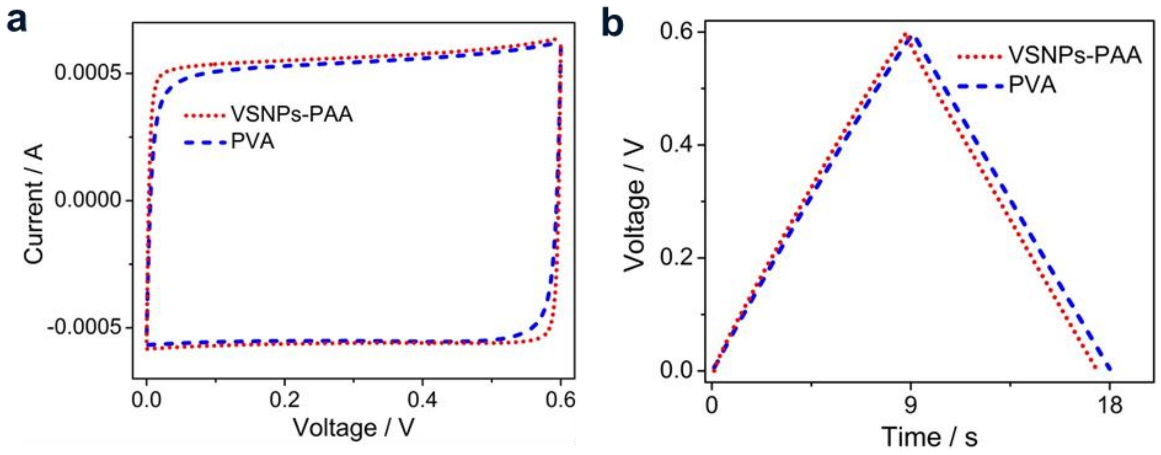

Supplementary Figure 7 | Electrochemical performances of supercapacitors with VSNPs-PAA and PVA as electrolytes. **a**, CV curves. **b**, GCD curves using VSNPs-PAA (red, dotted) and PVA (blue, dashed) as solid-state electrolytes at a constant mass ratio of polymer:H_3PO_4:H_2O. Both CV and GCD curves of PPy supercapacitors using VSNPs-PAA and common PVA electrolytes overlay completely, suggesting that VSNPs-PAA can be a perfect alternative to PVA as an electrolyte without compromising the electrode performance.

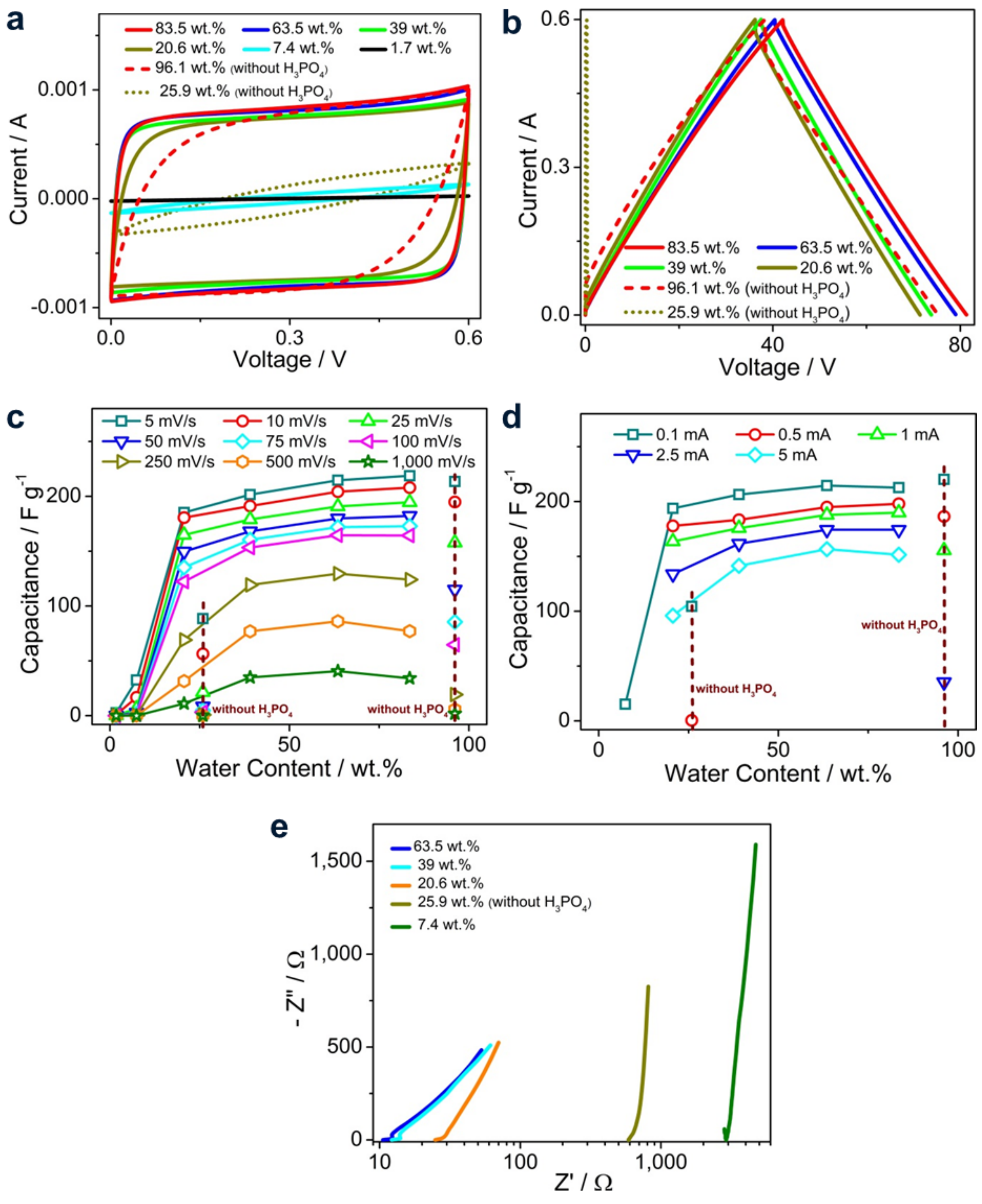

Supplementary Figure 8 | Electrochemical performances of supercapacitors using VSNPs-PAA electrolytes. a, CV at a scan rate of 25 mV/s with various water contents ranging 1.7 - 96.1 wt.% with and without H_3PO_4. **b**, GCD curves at a current of 0.5 mA with various water contents ranging 20.6 - 96.1 wt.% with and without H_3PO_4. **c**, Capacitances with respect to water content

under various scan rates. **d**, Capacitances with respect to water content under various charging/discharging currents. **e**, Nyquist plots of the facilely assembled supercapacitor with various water contents. Consistent with the results that ionic conductivity is affected by water content in the VSNPs-PAA polyelectrolyte, the CV (Supplementary Fig. 8a) and GCD (Supplementary Fig. 8b) curves are also remarkably different at various water contents. Both the CV and GCD curves get larger with increased water content in the VSNPs-PAA polyelectrolyte. Under all scan rates and charging/discharging currents, the capacitance increases with water content in the range of 1.7 to 96.1 wt.%, varying up to four orders of magnitude (Supplementary Fig. 8c and d). The enhanced capacitances can be attributed to high ion mobility at high water contents and convenient ion transfer in the moisturized electrolyte/electrode interface. Electrochemical impedance spectroscopy measurements (Supplementary Fig. 8e) also reflect these results. The supercapacitors exhibit small systematic resistance (the intercept at the Z`-axis) and small overall impedance (the end point in the Nyquist plot) at high water contents. The sufficiently extended polymer chains favor ion transportation in the electrolyte and at the electrolyte/electrode interface, thereby reducing the resistance and increasing the capacitance. In addition to water content, the protons that penetrate the VSNPs-PAA polyelectrolyte also contribute to capacitance enhancement. As observed in Supplementary Fig. 8a-e, the performance of VSNPs-PAA polyelectrolyte without H_3PO_4 penetration, though at higher water contents, is inferior. The difference in electrolytes with and without H_3PO_4 is more obvious at faster scan rates and higher charging/discharging currents, revealing the important role that transportation of available ions plays during the fast electrochemical dynamic process.

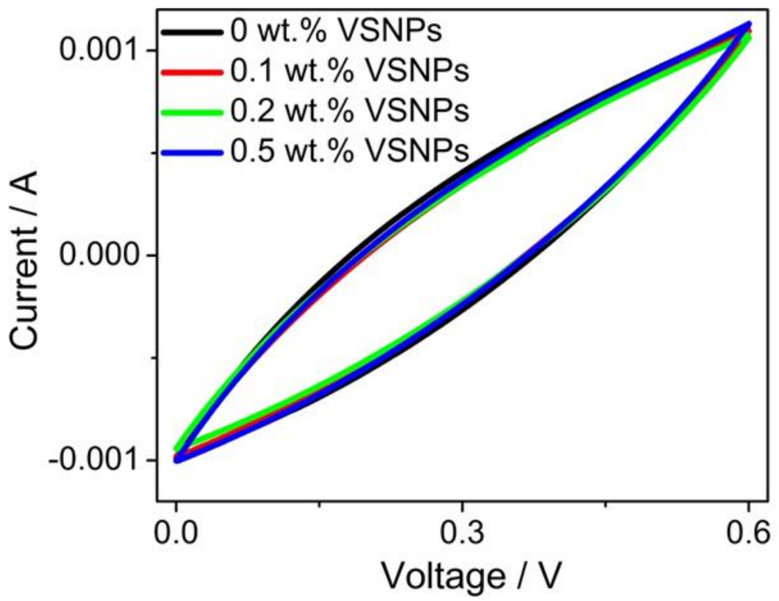

Supplementary Figure 9 | CV curves for VSNPs-PAA with various contents of VSNPs at a scan rate of 100 mV/s. The VSNPs content has no effects on electrochemical performance. As high density of PAA chains surround the VSNPs, ion transport is not affected by VSNPs directly. It is reasonable to obtain similar electrochemical performance using VSNP-PAA electrolytes at different VSNP content.

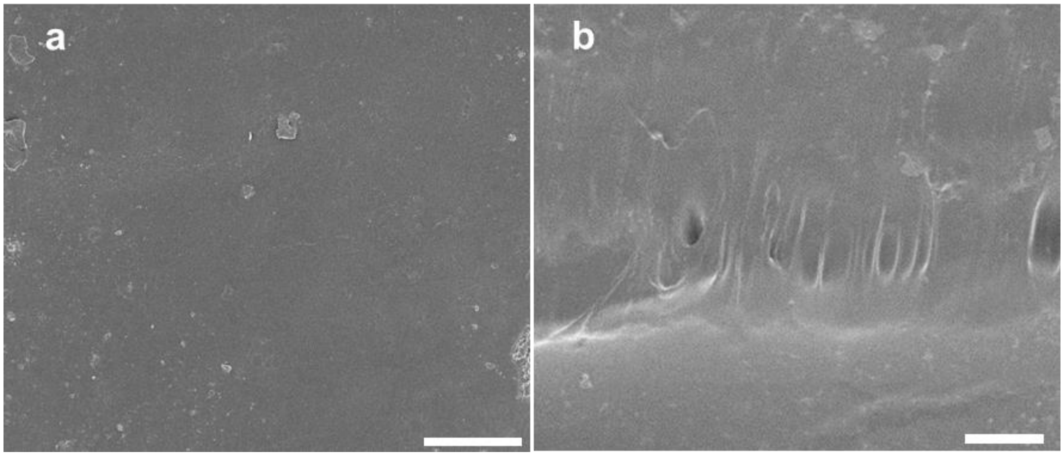

Supplementary Figure 10 | Characterization of VSNPs-PAA before and after healing. a, A scanning electron microscope (SEM) image of the pristine VSNPs-PAA polyelectrolyte. **b**, An SEM image of the VSNPs-PAA polyelectrolyte after healing. Both scale bars: 50 μm. The VSNPs-PAA polyelectrolyte is automatically healed. A scar can be observed. Its morphology indicates a well-bonded interface between the broken two pieces of VSNPs-PAA.

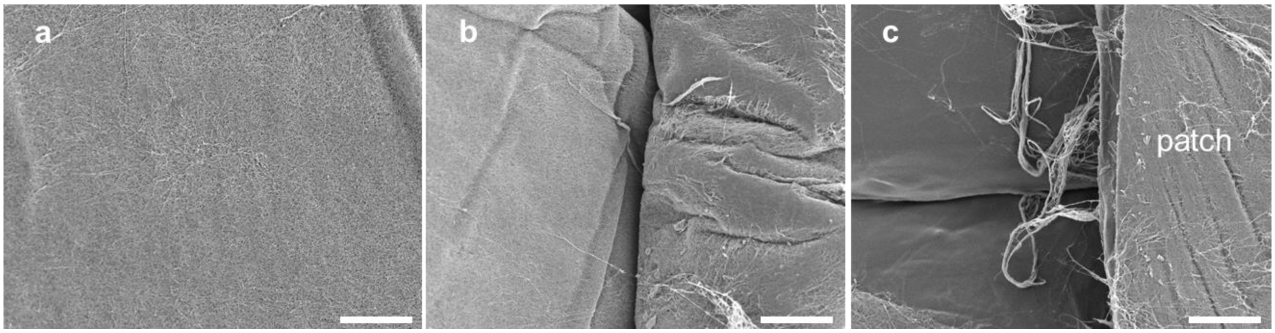

Supplementary Figure 11 | Characterization of VSNPs-PAA before and after healing. a, An SEM image of the PPy@CNT electrode. **b**, An SEM image of two manually aligned cut PPy@CNT electrodes, indicating a poor connection. **c**, An SEM image of the two cut PPy@CNT electrodes after electrical healing with a CNT patch. The patch bridges between the two PPy@CNT electrodes to realize an effective electronic connection. All scale bars: 10 µm. Obviously, there is a scar on the PPy@CNT electrode after cutting, which suggests a poor electronic connection between the two electrodes. This will greatly increase internal resistance of the supercapacitor. However, after the CNT patch is applied on the electrode scar, the electric connection is rebuilt between the two broken pieces of electrode. This is the key to achieving high self-healing efficiency for supercapacitor devices.

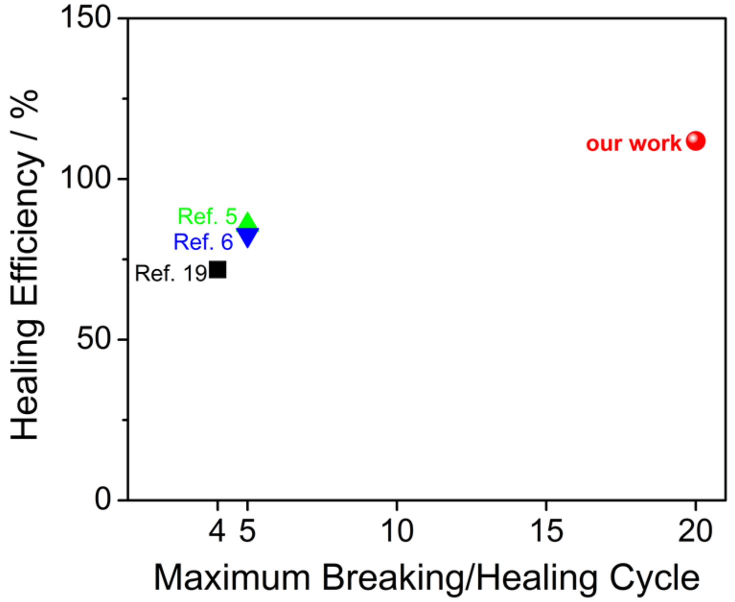

Supplementary Figure 12 | Comparison of healing efficiency at the maximum breaking/healing cycle of self-healable supercapacitors. Our self-healable supercapacitor can retain complete healing efficiency, maintaining capacitance within even 20 cycles of breaking/healing. Other literatures on self-healable supercapacitors only studied up to 5 cycles of breaking/healing, and 14.3%～28.2% of capacitance was lost after healing. Therefore, our approach provides an ultimate solution for self-healing solid-state supercapacitors.

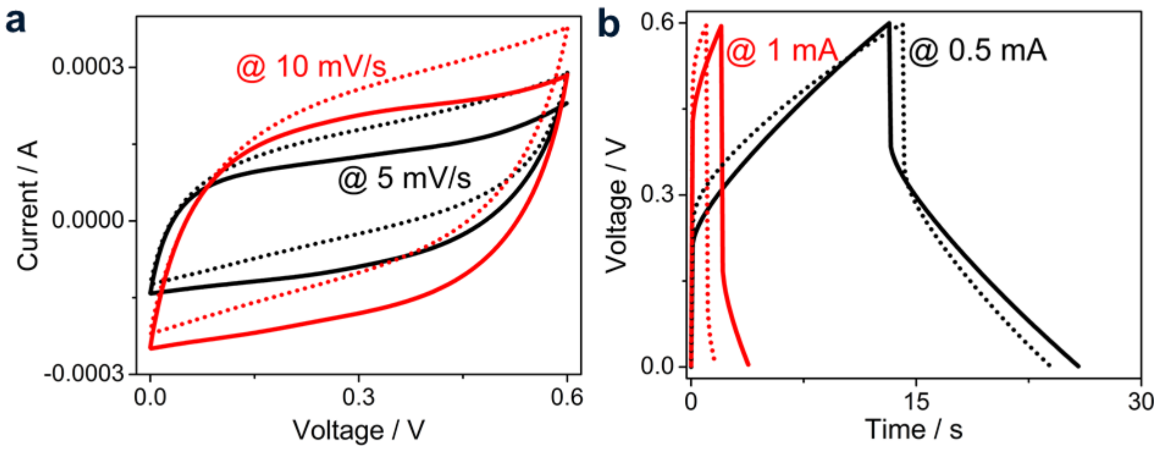

Supplementary Figure 13 | Electrochemical performances of the supercapacitor without a patch. a, CV curves. **b**, GCD curves of the supercapacitor without a patch before (solid) and after (dotted) the 1st breaking/healing cycle. The electrochemical performance of the supercapacitor without a patch deteriorates after merely one breaking/healing cycle, as revealed by the smaller CV loop, less discharging time, higher resistance, and distorted CV and GCD curves.

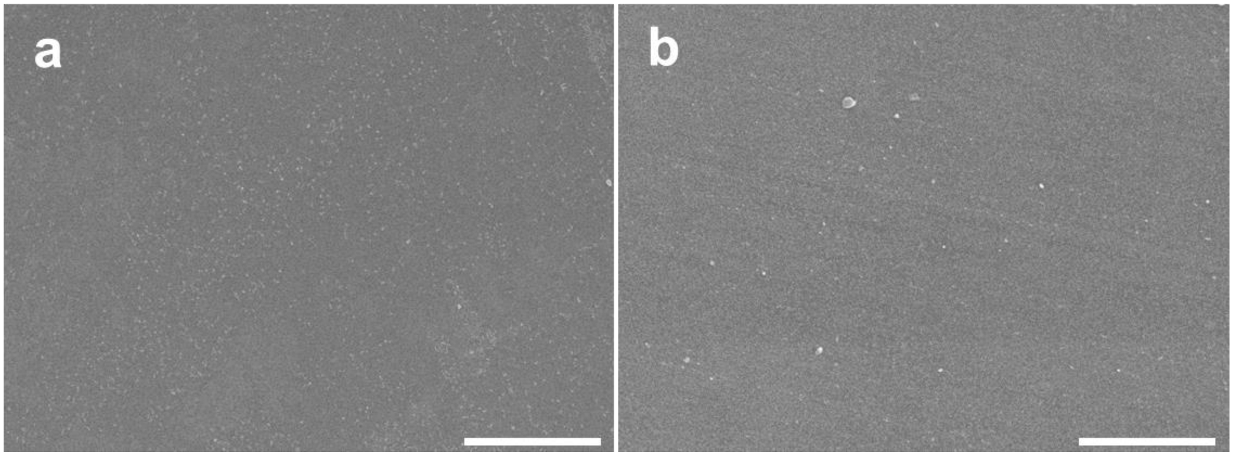

Supplementary Figure 14 | Characterization of VSNPs-PAA before and after stretch. a, An SEM image of the VSNPs-PAA polyelectrolyte before stretch. **b**, An SEM image of the VSNPs-PAA polyelectrolyte after stretch to a strain of 2000%. Both scale bars: 10 μm. The VSNPs-PAA electrolyte is non-directional before stretching. After being stretched to a high strain of 2000%, it becomes directional along the direction of stretch, as indicated by some fringes observed on the polyelectrolyte surface. This signifies that the molecular chains are stretched, as schematically represented in Figure 2d in the manuscript.

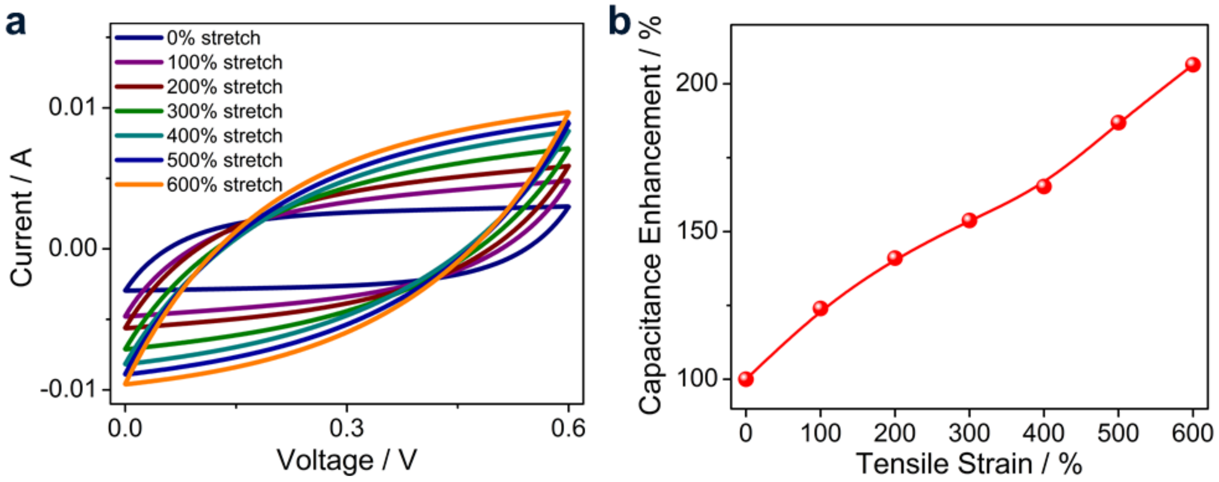

Supplementary Figure 15 | Electrochemical performances of the stretchable supercapacitor. **a**, CV curves under various tensile strains from 0% to 600% at a scan rate of 0.1 V/s. **b**, Capacitance enhancement ratios obtained from CV curves as a function of tensile strain. Consistent with GCD curves, these CV profiles expand with increased strain. The capacitance calculated from CV curves increases up to 2.1-fold at 600% strain.

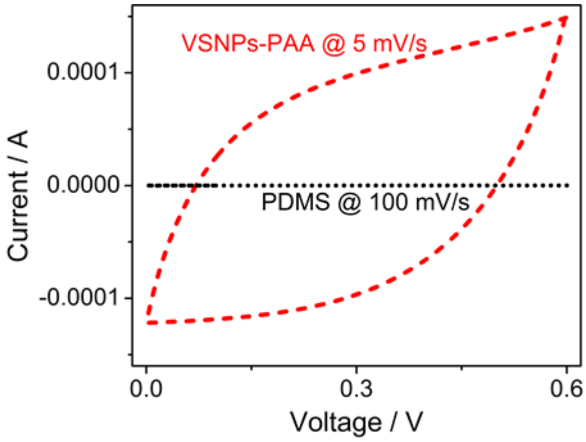

Supplementary Figure 16 | Comparative CV curves for supercapacitors using electrolytes of VSNPs-PAA (red, dashed) at a scan rate of 5 mV/s and PDMS (black, dotted) at a scan rate of 100 mV/s. Compared with VSNPs-PAA, there is no CV loop when PDMS is used as the electrolyte even at a high scan rate of 100 mV/s. This reveals that PDMS cannot serve as an electrolyte, albeit commonly used as a substrate material in super-stretchable devices.

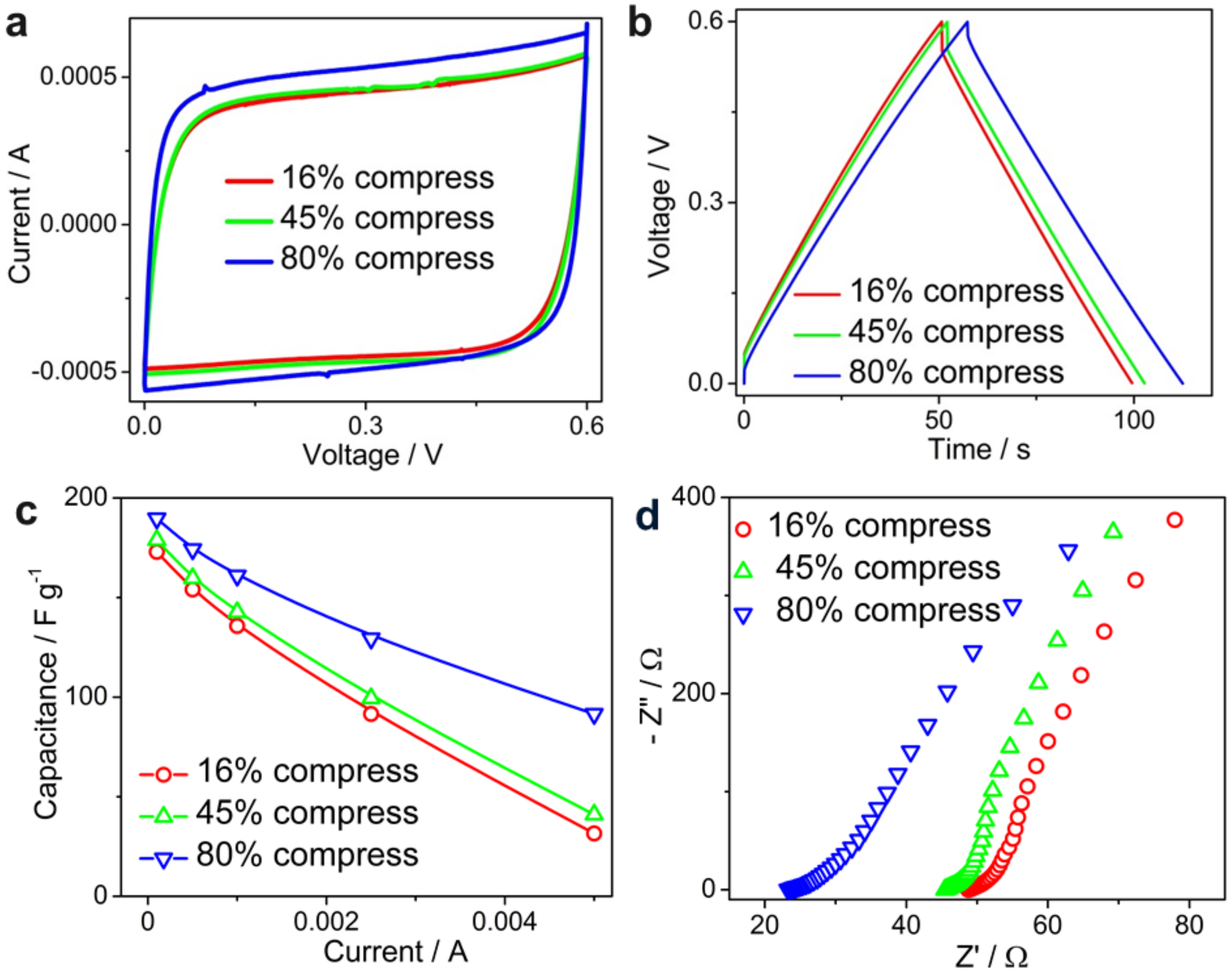

Supplementary Figure 17 | Electrochemical performances of the compressible supercapacitor. **a**, CV curves at a scan rate of 10 mV/s. **b**, GCD curves at a charging/discharging current of 0.5 mA. **c**, Capacitance as a function of the charging/discharging current. **d**, Nyquist plots of the supercapacitor under various compressive strains. Similar to the case of applied stretch, electrochemical performance also increases with compressive strain (Supplementary Fig. 17a-c). The pressure-improved interfacial contact between the electrolyte and the electrodes is the main reason for the improved performance under compression. As seen in Supplementary Fig. 17d, the supercapacitor has a smaller systematic resistance (the intercept at the Z`-axis) at a higher compressive strain. This result is further confirmed by the decreased *IR* drop shown in Supplementary Fig. 17b. With increased compressive strain, *IR* drop decreases. This suggests smaller systematic resistance, and thus, improved contact at the interface of the electrolyte/electrode under compression. Such compress-induced capacitance improvement is consistent with many studies on supercapacitors under pressure[5, 6]. To summarize, the

compressive stress benefits the interfacial contact and, therefore, the ion transfer from the electrolyte to the surface of electrode, which contributes to the higher capacitance.

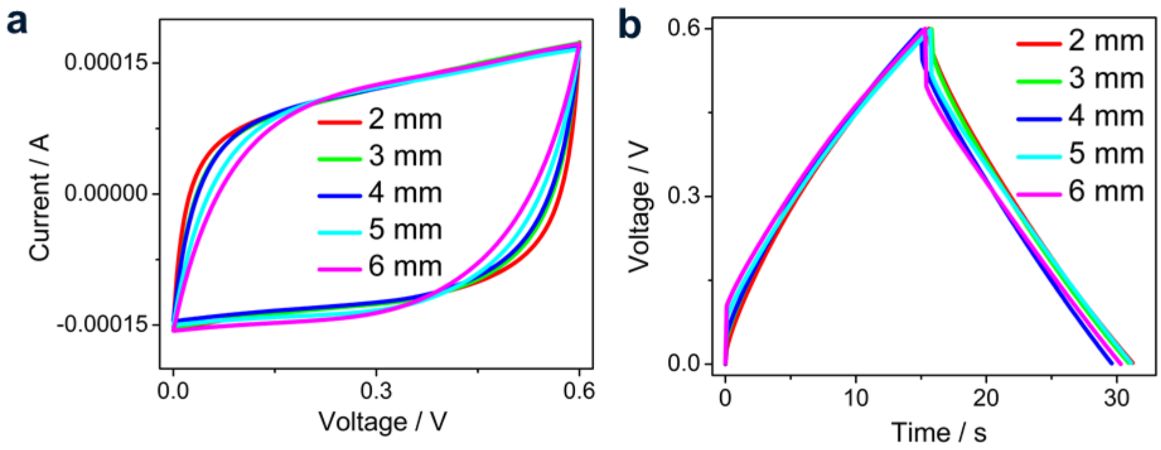

Supplementary Figure 18 | Electrochemical performances of supercapacitors with various electrolyte thicknesses. a, Comparative CV curves. **b**, GCD curves of supercapacitors with various VSNPs-PAA electrolyte thicknesses. In the range of electrolyte thicknesses we studied, the performance of supercapacitors was not affected. Thus, the effect of electrolyte thickness on the capacitance can be ignored in our devices during compression.

Supplementary Note 1

The very facile fabrication of self-healable supercapacitors is demonstrated in Supplementary Movie 1: Self-heal the supercapacitor. The entire repairing process simply brings the cut interfaces together, paving two small patches of CNT paper on the wounds at room temperature. The repaired supercapacitor successfully powers an LED bulb. **S**upplementary Movie 2 (Stretch the supercapacitor) shows the process of stretching the supercapacitor. The supercapacitor is easily stretched to high degree, meanwhile still lighting an LED bulb.

Supplementary Methods

Tensile strength of VSNPs-PAA polyelectrolyte samples (cylindrical with diameter 5 mm) was measured by a mechanical testing system (Zwick-Roell Z005, Ulm, Germany) at a strain rate of 100 mm min^{-1}. The microstructure and morphology of electrodes were characterized by scanning electron microscope (SEM) (JEOL JSM-6335F) with an acceleration voltage of 5 kV. The particle size of VSNPs was observed by transmission electron microscope (TEM) (Hitachi H7700) at an acceleration voltage of 100 kV. FTIR (AVATAR 380) was used to visualize vinyl groups on the as-synthesized VSNPs. Raman spectra were obtained by RENISHAW Raman microscope (RA100) with an excitation wavelength of 633 nm. For the compressible supercapacitor evaluation, different weights (100 g, 280 g, and 500 g) were placed on the supercapacitor to impose compression during electrochemical measurement. To confirm the hydrogen bonding effect on the self-healing of VSNPs-PAA polyelectrolyte, several droplets of urea solution (1 M) were dropped on the surface of the wound in order to break the hydrogen bonding at the interface.

Supplementary References

1. Liu, J., Song, G., He, C. & Wang, H. Self-healing in tough graphene oxide composite hydrogels. *Macromol. Rapid Commun.* **34**, 1002-1007 (2013).

2. McQueen-Mason, S. & Cosgrove, D. Disruption of hydrogen bonding between plant cell wall polymers by proteins that induce wall extension. *Proc. Natl. Acad. Sci.* **91**, 6574-6578 (1994).

3. Xia, H., Wang, Y., Lin, J. & Lu, L. Hydrothermal synthesis of MnO_2/CNT nanocomposite with a CNT core/porous MnO_2 sheath hierarchy architecture for supercapacitors. *Nanoscale Res. Lett.* **7:33**, 1-10 (2012).

4. Wang, J., Xu, Y. L., Yan, F., Zhu, J. B. & Wang, J. P. Template-free prepared micro/nanostructured polypyrrole with ultrafast charging/discharging rate and long cycle life. *J. Power Sources* **196**, 2373-2379 (2011).

5. Liu, X. H., Wu, D. B., Wang, H. L. & Wang, Q. G. Self-recovering tough gel electrolyte with adjustable supercapacitor performance. *Adv. Mater.* **26**, 4370-4375 (2014).

6. Masarapu, C., Wang, L. P., Li, X. & Wei, B. Q. Tailoring electrode/electrolyte interfacial properties in flexible supercapacitors by applying pressure. *Adv. Energy Mater.* **2**, 546-552 (2012).
